# A novel dual-targeting strategy to suppress cariogenic bacteria and biofilms with engineered ARG/FBG bioactive composites

**DOI:** 10.1093/rb/rbag058

**Published:** 2026-03-20

**Authors:** Xili Qiu, Tongjun Li, Jincai Guo, Hua Liu, Jing Liu, Xiaojing Chen

**Affiliations:** Hunan Key Laboratory of Oral Health Research & Xiangya Stomatological Hospital & Xiangya School of Stomatology, Central South University, 72 Xiangya Road, Changsha, Hunan, 410008, China; School of Life Sciences, Central South University, 172 Tongzipo Road, Changsha, Hunan, 410083, China; School of Stomatology, Hunan University of Chinese Medicine, 389 Youyi Road, Changsha, Hunan, 410004, China; Changsha Stomatological Hospital, 389 Youyi Road, Changsha, Hunan, 410004, China; School of Stomatology, Hunan University of Chinese Medicine, 389 Youyi Road, Changsha, Hunan, 410004, China; Changsha Stomatological Hospital, 389 Youyi Road, Changsha, Hunan, 410004, China; School of Life Sciences, Central South University, 172 Tongzipo Road, Changsha, Hunan, 410083, China; School of Life Sciences, Central South University, 172 Tongzipo Road, Changsha, Hunan, 410083, China; FuRong Laboratory, 88 Xiangya Road, Changsha, Hunan, 410008, China; Hunan Key Laboratory of Oral Health Research & Xiangya Stomatological Hospital & Xiangya School of Stomatology, Central South University, 72 Xiangya Road, Changsha, Hunan, 410008, China; FuRong Laboratory, 88 Xiangya Road, Changsha, Hunan, 410008, China

**Keywords:** caries prevention, arginine, fluoride-containing bioactive glass, ARG/FBG composites, *Streptococcus mutans*

## Abstract

Dental caries is a biofilm-mediated disease primarily driven by *Streptococcus mutans* (*S. mutans*), which ferments dietary sugars to produce acids that demineralize enamel. This study developed a novel composite combining L-arginine (ARG) and fluoride-containing bioactive glass (FBG) to achieve dual antibacterial and antibiofilm effects. The ARG/FBG composite (1:1 mass ratio) was assessed *in vitro* against *S. mutans*. Antibacterial activity was quantified by AlamarBlue assay and colony-forming unit (CFU) counts, while antibiofilm efficacy was evaluated using scanning electron microscopy and confocal laser scanning microscopy. ARG inhibited planktonic growth but showed limited biofilm disruption, whereas FBG effectively disrupted biofilm architecture without affecting planktonic growth. The composite (7.5 mg/mL ARG + 7.5 mg/mL FBG) demonstrated enhanced complementary action, achieving near-complete eradication of planktonic cells (∼6-log_10_ CFU reduction at 24 h) and profound biofilm destruction. TEM-EDS revealed intracellular co-localization of fluoride, calcium and phosphorus within treated bacteria. RNA sequencing and RT-qPCR showed downregulated key virulence genes (*ldh* and *luxS*) and disrupted metabolic pathways related to carbon utilization and acid production. The composites exhibited favorable biocompatibility. These findings indicate that ARG/FBG bioactive composites potently suppress *S. mutans* through biofilm disruption, metabolic inhibition and potential *in situ* mineralization, offering a promising strategy for caries prevention.

## Graphical abstract

**Graphical Abstract:**
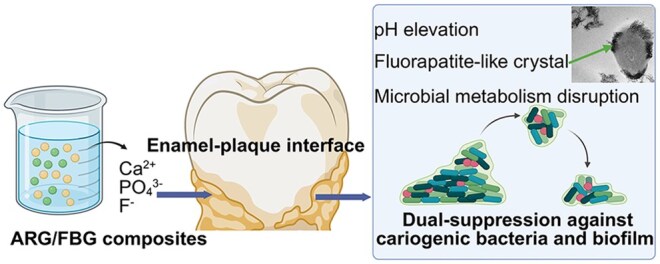

## Introduction

Dental biofilms arise from oral microbiome dysbiosis [1], where carbohydrate fermentation drives biofilm acidification, enamel demineralization and enrichment of aciduric and acidogenic species [2]. *Streptococcus mutans* (*S. mutans*), a keystone cariogenic pathogen, promotes biofilm pathogenicity via extracellular polymeric substances (EPS) synthesis and sustained acid production [3–6]. Accordingly, strategies that create alkaline microenvironments and selectively attenuate *S. mutans* virulence while preserving microbial homeostasis are particularly attractive for biomaterial-based caries prevention.

Current caries-preventive materials remain limited in their efficacy. Fluoride, a cornerstone of caries prevention, promotes fluorapatite formation and inhibits bacterial glycolysis [7–11], yet its long-term efficacy is limited by insufficient activity against mature biofilms, reduced bioavailability and fluorosis risk [12–14]. Arginine (ARG) has emerged as a promising adjunct, with epidemiological studies linking elevated salivary ARG levels and arginine deiminase system (ADS) to reduced caries incidence [15, 16]. Several commensal streptococci metabolize ARG via ADS to generate ammonia [17, 18], thereby raising biofilm pH [9, 19, 20]. ARG supplementation promotes a commensal-dominated microbiota and mitigates ecological drivers of caries pathogenesis [21]. However, the antibacterial effect of ARG against *S. mutans* is dose-dependent [22, 23]. Notably, combining ARG with fluoride enhanced remineralization and modulated biofilm ecology [9, 24–28], but high-ARG/fluoride varnish shows potential cytotoxicity [29], underscoring the need to optimize this combination.

Bioactive glasses such as 45S5 release calcium and phosphate ions to support remineralization, yet their antibacterial activity remains modest [30, 31]. Qiu *et al.* demonstrated that combining ARG with fluoride-containing bioactive glass (FBG, 6.0 mol% CaF_2_) synergistically enhanced the anti-demineralization effect on enamel, attributed to fluorapatite formation and possible interfacial ARG–F–Ca complexes [32]. Similarly, the bioavailability of ARG and fluorine was improved when co-administered with FBG [33, 34]. The ARG/FBG bioactive materials integrate ecological modulation with mineral-supportive functions, offering a multifunctional platform for caries prevention.

While meta-transcriptomics and RNA-sequencing (RNA-seq) analysis enable comprehensive, high-resolution investigation of biofilm gene expression and functional pathways [35–38], the transcriptomic impact of ARG/FBG composites on *S. mutans* remains unknown and will be explored in this study.

Despite these advances, the antibacterial properties of ARG/FBG composites against *S. mutans* remain largely unexplored. We hypothesize that ARG and FBG act complementarily: ARG targets planktonic growth and modulates pH, while FBG disrupts the biofilm matrix and provides sustained ion release. Their combination in a bioactive composite may thus achieve superior dual-pathway suppression of *S. mutans* virulence. This study aims to use an *in vitro* simulated biofilm model to investigate the antibacterial, antibiofilm efficacy and the underlying mechanisms of ARG/FBG bioactive composites against *S. mutans*.

## Materials and methods

### Microbial culture and preparation of ARG/FBG composites

*S. mutans* (ATCC 25175) was obtained from China General Microbiological Culture Collection Center (CGMCC, China). The bacteria were cultured to mid-exponential phase in Brain-Heart Infusion (BHI, Oxoid, American) broth at 37°C under anaerobic conditions (85% N_2_, 10% H_2_, 5% CO_2_) for subsequent experiments. The linear relationship between *S. mutans* suspension concentration (CFU/mL, *y*) and optical density (OD, *x*) at 600 nm (*y* = 142.57*x* − 16.26, *R*^2^ = 0.9981) was determined using the dilution plating method.

FBG (nominal composition in mol%: 35.9 SiO_2_–52.2 CaO–5.9 P_2_O_5_–6.0 CaF_2_) was synthesized via a melt-quench method in an electronic furnace (Carbolite Gero, UK) at 1530°C for 1 h, as previously described [39]. FBG with particle size less than 38 μm and L-arginine (ARG, A8094, Sigma, USA) were dissolved in the BHI broth to evaluate their antimicrobial efficacy. ARG and FBG were combined at a 1:1 mass ratio to prepare the ARG/FBG composites. BHI broth alone served as the control. The antibacterial and antibiofilm effects of ARG/FBG bioactive composites were compared with their individual components’ groups and the negative control group.

### Determination of MIC

The minimum inhibitory concentrations (MICs) of ARG and FBG against *S. mutans* were determined using the broth microdilution method [40, 41]. *S. mutans* cultures were incubated in BHI broth on an orbital shaker (100 rpm) for 24 h at 37°C under 5% CO_2_. Optical density at 600 nm (OD_600_) was recorded at 0 and 24 h (Bio Tek, USA). ARG and FBG were subjected to 2-fold serial dilutions in BHI broth, with concentrations ranging from 60 to 0.9375 mg/mL. The MIC was defined as the lowest concentration of the composite that resulted in bacterial suspensions with no visible cells and an OD_600_ difference (OD_600_ 24 h − OD_600_ 0 h) of less than 0.05. All tests were performed in two independent biological replicates, each with three technical replicates.

### Cell viability assay

FBG has been shown to release the majority of its constituent ions within 24 h of immersion, as previously described [39]. In this study, extracts were prepared by incubating ARG, FBG or their mixture in α-MEM medium for 24 h, followed by sterile filtration (0.22 µm). Specifically, 75 mg of powder was added to 50 mL of α-MEM and incubated for 24 h. The extracts were supplemented with 10% (v/v) fetal bovine serum, 1% (v/v) penicillin/streptomycin and 1% (v/v) glutamine [42].

Fibroblasts and pre-osteoblastic (MC3T3-E1) cells (Shanghai cell bank, ATCC, China) were routinely cultured in supplemented α-MEM (37°C, 5% CO_2_), until reaching 80–90% confluency. For viability assays, cells were seeded in 96-well plates at a density of 1 × 10^3^ cells/well and incubated overnight under standard culture conditions. Cells were then treated with the prepared extracts for 1, 4 and 7 days. Extracts without ARG or FBG served as the treatment control group, extracts with no powder served as the negative control group. Media were refreshed every 48 h.

Cell viability was measured using a CCK-8 kit (Bio sharp, China). After 2 h incubation with the CCK-8 reagent, the absorbance was recorded at 450 nm using a microplate reader. All experiments were conducted in three independent biological replicates, each with four technical replicates.

### Antibacterial efficacy

The inhibitory effects of ARG/FBG composites on the growth of planktonic *S. mutans* were assessed using the AlamarBlue assay (Bio-Rad, USA) and colony-forming unit (CFU) counting.

ARG and FBG were subjected to 2-fold serial dilutions in BHI broth to final concentrations ranging from 60 to 1.875 mg/mL in 96-well plates. *S. mutans* suspensions were added, and samples were incubated on an orbital shaker (100 rpm, 37°C, 5% CO_2_) for 2, 4, 6 h. After incubation, AlamarBlue reagent was added, and the plates were incubated for an additional 30 min. The reaction mixtures were centrifuged at 4500 rpm for 5 min. The supernatants were transferred to a fresh 96-well plate, and absorbance was measured at 570 and 600 nm. Assays were performed in triplicate across three independent experiments. The inhibition rate was calculated according to the manufacturer’s instructions as the following formula:


$$\mathrm{Antibacterial}\;\mathrm{rate}\;(\%)=(1-\frac{\left(117216\times A1)-(80586\times A2\right)}{\left(117216\times P1)-(80586\times P2\right)})\times 100\%$$

*A*1 = OD_570_ (treatment), *A*2 = OD_600_ (treatment), *P*1 = OD_570_ (control), *P*2 = OD_600_ (control).

To evaluate the long-term antibacterial activity, *S. mutans* suspensions in BHI broth were treated with ARG and FBG (at concentrations from 30 to 7.5 mg/mL) and incubated for 6 or 24 h under the same conditions. Samples were serially diluted (7–9 steps of 10-fold each) in BHI broth, and 100 µL aliquots were spread on the BHI agar. Plates were incubated anaerobically at 37°C with 5% CO_2_ for 48 h before imaging. Experiments were performed in triplicate. CFU data were log-transformed for statistical analysis.

Based on these results, the 7.5 mg/mL ARG + 7.5 mg/mL FBG composite demonstrated significant antibacterial activity against planktonic *S. mutans* at all tested time points (2–24 h). Accordingly, subsequent experiments employed the following treatment groups to further assess antibacterial mechanisms: control (no treatment), 15ARG (15 mg/mL ARG), 15FBG (15 mg/mL FBG), 15ARG + 15FBG (15 mg/mL ARG + 15 mg/mL FBG) and 7.5ARG + 7.5FBG (7.5 mg/mL ARG + 7.5 mg/mL FBG). In these groups, 15ARG and 15FBG served as the single-treatment controls, 15ARG + 15FBG and 7.5ARG + 7.5FBG groups were the experimental groups.

### Antibiofilm effect

The inhibitory effect on biofilm formation was assessed using scanning electron microscope (SEM). Biofilms were cultured aerobically in BHI broth supplemented with ARG and FBG at 37°C (5% CO_2_) for either 6 or 24 h. Following incubation, the biofilms were fixed with 2.5% glutaraldehyde at 4°C overnight. Samples were then dehydrated through a graded ethanol series (40%, 60%, 70%, 80%, 85%, 90%, 100%; 15 min per step), air dried for 48 h and sputter-coated with gold. Triplicate samples from each group were imaged using SEM (ESCAN MIRA4 LMH, Czech Republic) operating at 5 keV.

The biofilm disruption effect of ARG/FBG composites was observed via Confocal laser scanning microscopy (CLSM). *S. mutans* biofilms were formed statically in laser confocal petri dishes using BHIS broth (BHI supplemented with 1% w/v sucrose) for 24 h at 37°C under 5% CO_2_. ARG and FBG (dispersed in 1 mL BHIS) were then added, and the cultures were incubated for an additional 12 h. After rinsing with PBS, biofilms were stained in the dark for 30 min with SYTO 9 (*λ*_481nm_/*λ*_540nm_) and propidium iodide (*λ*_591nm_/*λ*_700nm_) from the Live/Dead™ Bac Light™ Bacterial Viability Kit (Invitrogen, USA). CLSM imaging was performed using a Zeiss system (Germany) equipped with a 40× oil-immersion objective. Z-stack images were acquired with 1 μm step size and reconstructed in 3D. Bacterial vitality (green: live/red: dead ratio) and biofilm density were assessed from three random fields per sample (*n* = 3 independent biofilms).

### TEM and TEM-EDS

*S. mutans* suspensions (10^8^ CFU/mL) were treated with ARG and FBG in BHI broth and incubated at 100 rpm, 37°C, 5% CO_2_ for 4 h. Samples were pelleted by centrifugation (6000 rpm, 5 min) and fixed in 2.5% glutaraldehyde at 4°C for 12 h, then post-fixed in 1% osmium acid for 2 h. Samples were dehydrated using a graded acetone series (50–100%, 15 min per step), followed by embedding for 12 h. Ultrathin sections (50–100 nm) were cut using an ultramicrotome and double-stained with 3% uranium acetate–lead nitrate. Transmission electron microscopy (TEM) imaging was carried out using an instrument from FEI Company (USA), and elemental mapping was conducted using energy dispersive X-ray spectroscopy (EDS, JEOL f-200, Japan). For EDS, unstained sections mounted on copper grids were analysed.

### Transcriptomic profiling

RNA-seq analysis and quantitative reverse transcription polymerase chain reaction (RT-qPCR) were employed to elucidate the mechanism of ARG/FBG composites against *S. mutans*. *S. mutans* were treated with ARG and FBG for 1 h at 100 rpm, 37°C, 5% CO_2_. Cells from triplicate samples per group were harvested by centrifugation (400 rpm, 1 min, 4°C) to pellet and remove FBG particles. Total RNA was extracted using RNeasy Mini Kit (QIAGEN, Germany) with on-column DNase digestion. RNA integrity was confirmed using the Bioanalyzer 2100 (Agilent Technologies, USA). RNA libraries were prepared via fragmentation (94°C, 15 min), end-repaired/A-tailing, adapter ligation, size selection, PCR amplification, and purification. Library was quality-controlled using the Agilent TapeStation 4200 and quantified by qPCR (Applied Biosystems, USA). RNA-seq analysis was performed on an Illumina HiSeq instrument (Novogene, China). Raw reads were demultiplexed (bcl2fastq v2.17) and aligned to the *S. mutans* UA159 reference genome (RefSeq: GCF_019048645.1) using HISAT2. Differentially expressed genes (DEGs) were identified using the limma R package (|log_2_FC| > 0.5, *P *< 0.05). Functional analysis included principal component analysis (DESeq2 R, plotPCA), volcano plot generation (VolcaNoseR), GO enrichment analysis (GOseq, FDR < 0.05), KEGG pathway enrichment (KOBAS 3.0), and WGCNA-based co-expression network construction.

RT-qPCR was performed with three independent biological triplicates and three technical replicates. *S. mutans* exposed to ARG and FBG for 1 h were collected for RT-qPCR. Cells were pelleted (8000 rpm, 10 min) and lysed with lysozyme. Total RNA was extracted using TRIzol reagent as described previously [43, 44]. The extracted RNA was assessed for concentration and purity spectrophotometrically (*A*_260_/*A*_280_ > 1.8) and then reverse-transcribed using the HiScript Q RT SuperMix (Vazyme, China). The primers of *S. mutans* performed for RT-qPCR analysis are shown in Table 1. PCR was performed using ChamQ Universal SYBR qPCR Master Mix (Vazyme, China). Amplification was conducted on a QuantStudio 5 system using the following protocol: 95°C for 30 s, followed by 40 cycles of 95°C for 10 s and 60°C for 30 s. Gene expression was normalized to 16S rRNA and analysed using the 2^−ΔΔ^^*CT*^ method.

**Table 1:** RT-qPCR primers for *S. mutans* genes.

Genes	F	R
*16S rRNA*	CCTACGGGAGGCAGCAGTAG	CAACAGAGCTTTACGATCCGAAA
*ldh*	ACTTCACTTGATACTGCTCGTT	AACACCAGCTACATTGGCATGA
*luxS*	GCTTTGATGACTGTGGCTATTTG	ACTGCAGGCCTTCATACTATTG

### Statistical analysis

Data are presented as mean ± SD. Multi-group comparisons used one-way ANOVA with Tukey’s *post hoc* test, and the comparisons between two groups used paired *t*-tests. Significance thresholds were set as: *P* < 0.05 (*), <0.01 (**), <0.001 (***). Superscript letters (a–e) indicate significant differences among the treatment groups (*P *< 0.05). All statistical analyses were performed using GraphPad Prism 9.5.3.

## Results

### The MIC and biocompatibility of ARG/FBG composites

The MIC values of ARG, FBG and their composites against *S. mutans* are presented in Supplementary Figure S1. Both ARG and FBG individually exhibit MIC values of 15 mg/mL, matching that of the ARG/FBG composite at 7.5 mg/mL ARG + 7.5 mg/mL FBG. Detailed numerical data are available in Supplementary Table S1.

**Figure 1:**
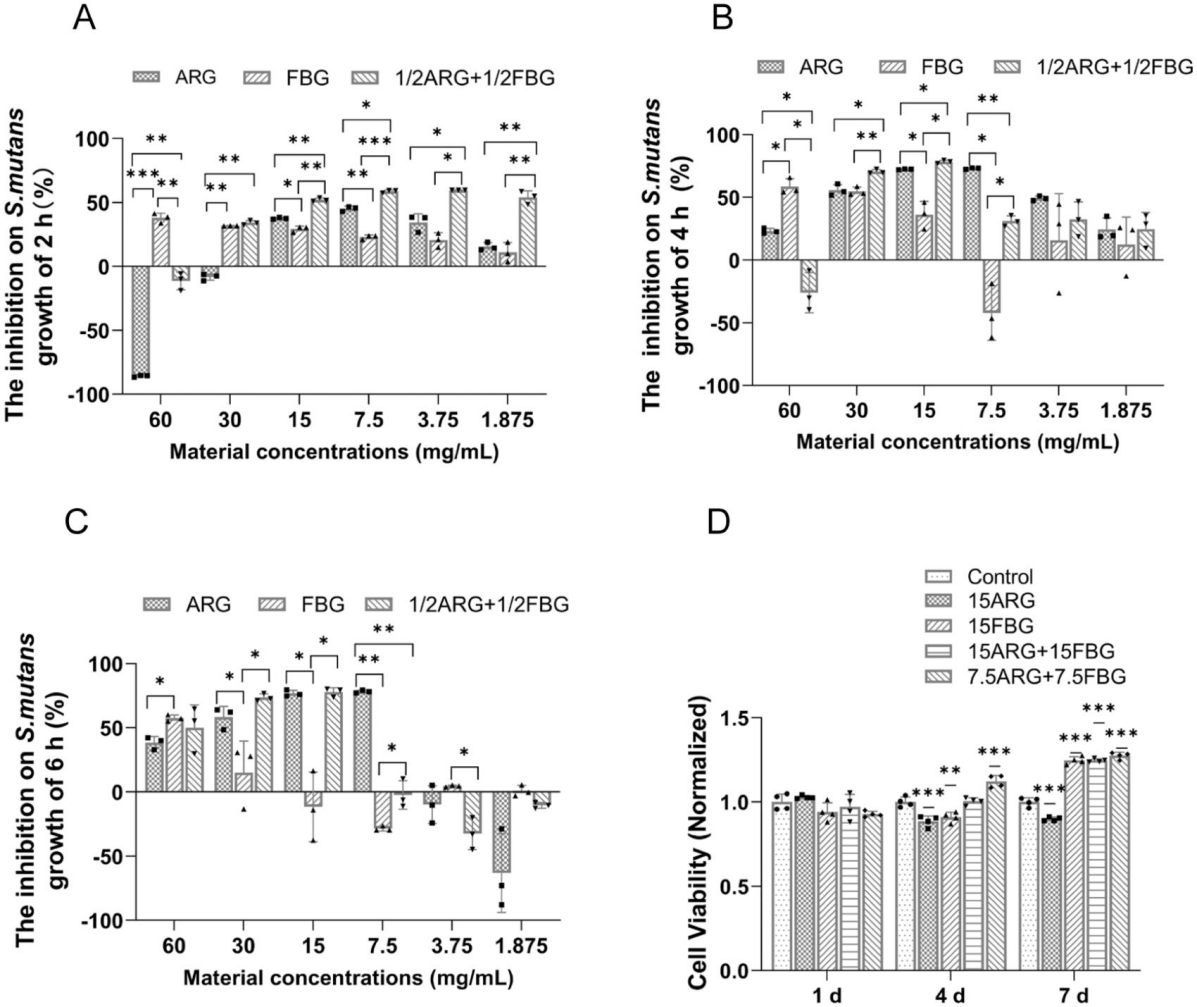
Antibacterial effects of ARG and FBG on planktonic *S. mutans* via AlarmarBlue assay at 2 h (**A**), 4 h (**B**) and 6 h (**C**). (**D**) Viability of fibroblasts exposed to ARG and FBG extracts normalized to the control, *P < 0.05, **P < 0.01 and ***P < 0.001 versus control.

The cytocompatibility of ARG, FBG and their composites was evaluated using the CCK-8 assay in fibroblasts and MC3T3-E1 pre-osteoblasts after 1, 4 and 7 days of exposure. As shown in Figure 1D and Supplementary Figure S2, all the treatment groups either maintained or slightly enhanced cell viability compared to the control. These findings suggest excellent biocompatibility of the tested materials.

### ARG/FBG composites enhanced the antibacterial properties against *S. mutans*

The inhibitory activity of ARG, FBG and their composites on planktonic *S. mutans* was evaluated using AlamarBlue and CFU assays at 2, 4 and 6 h. As shown in Figure 1A–C, ARG at both 15 and 7.5 mg/mL significantly inhibited bacterial growth at all time points (∼40% at 2 h, ∼65% at 4 h, ∼70% at 6 h). In contrast, FBG alone exhibited limited antibacterial activity, even at 30 mg/mL. The composite of 7.5ARG + 7.5FBG exhibited enhanced antibacterial effects compared to the individual components (*P *< 0.05) upon treatment. Notably, the 7.5ARG + 7.5FBG composite showed potent inhibition at all time points (∼50% at 2 h, ∼70% at 4 h, ∼80% at 6 h), and the effect was similar to that of the higher-dose 15ARG + 15FBG group. The 3.75ARG + 3.75FBG composite also showed enhanced antibacterial activity compared to single components at 2 h, although its efficacy declined by 4 and 6 h.

**Figure 2:**
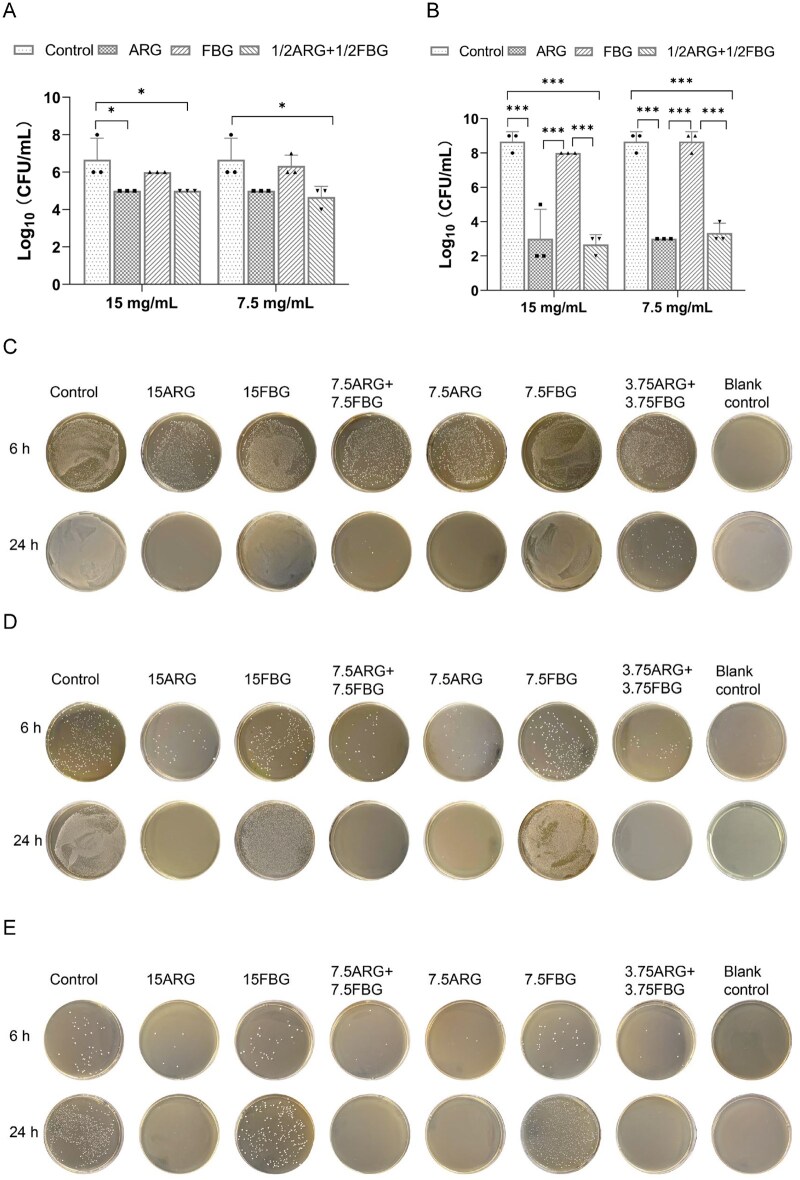
Antibacterial effects of ARG and FBG on planktonic *S. mutans* via CFU counts. (**A, B**) Quantification of bacterial viability after 6 h (**A**) and 24 h (**B**) treatments. (**C–E**) Representative CFU images for selected treatment groups, (**C**) 10^2^-fold dilution plates, (**D**) 10^4^-fold dilution plates, (**E**) 10^6^-fold dilution for 6 h and 10^7^-fold dilution for 24 h.

Quantitative CFU analysis after 6 and 24 h treatment is shown in Figure 2A and B, with representative colony images in Figure 2C–E. FBG (7.5 and 15 mg/mL) showed negligible activity compared to the control, while the 15ARG and 7.5ARG groups demonstrated significantly greater inhibition. Interestingly, the efficacy of ARG increased over time (with CFU counts of ∼5-log_10_ at 6 h and ∼3-log_10_ at 24 h), while the CFU counts in the control group steadily rose (from ∼7-log_10_ to ∼9-log_10_ CFU over the same period). Importantly, the 7.5ARG + 7.5FBG composite achieved comparable antibacterial effects to 15ARG alone at both 6 and 24 h, while the 3.75ARG + 3.75FBG composite was less effective than the higher-dose composites, indicating the potential of the 7.5ARG + 7.5FBG composite as a low-dose but effective antimicrobial strategy.

### ARG/FBG composites potently suppressed the biofilm of *S. mutans*

**Figure 3:**
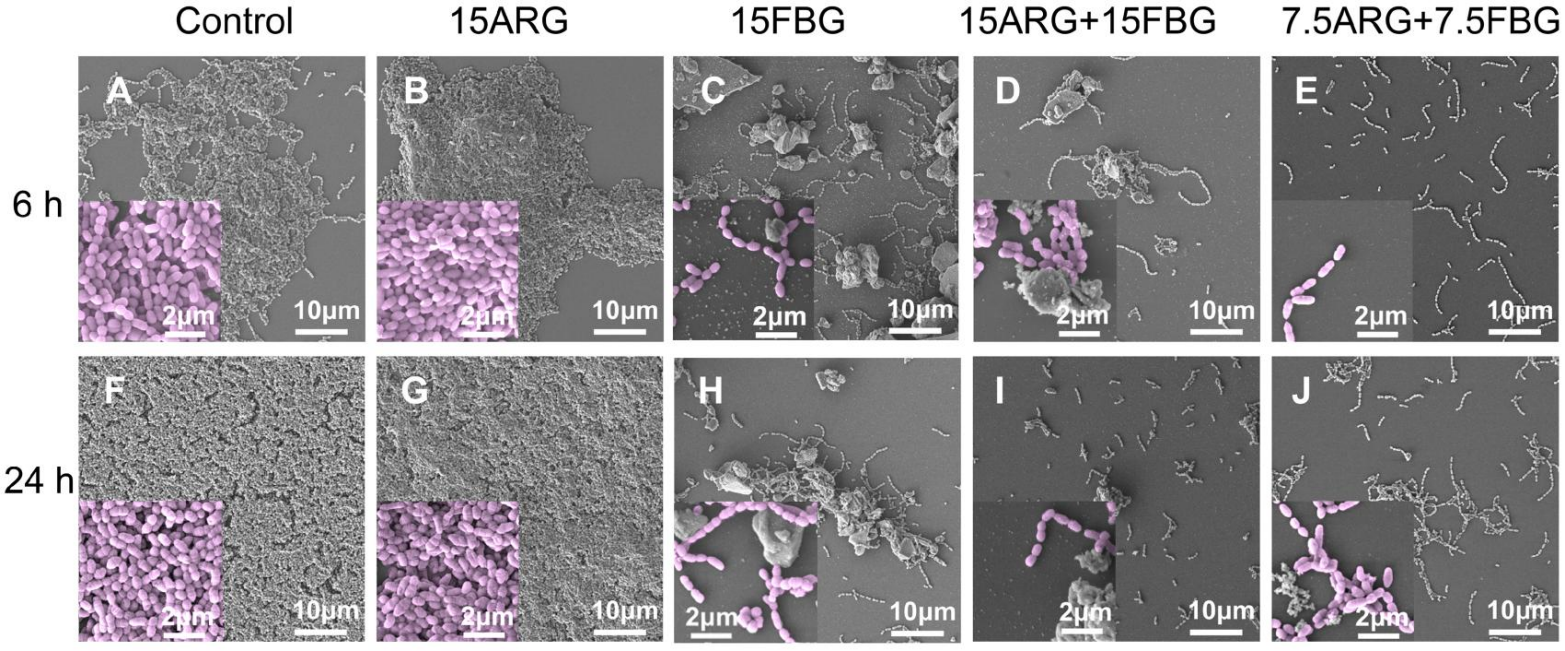
SEM characterization of *S. mutans* biofilms following 6 h (**A–E**) and 24 h (**F–J**) treatment. Images acquired at 5000× (scale bar = 10 μm) and 20 000× (scale bar = 2 μm) magnification.

SEM images revealed distinct differences in biofilm architecture following 6 and 24 h treatments with various formulations (Figure 3). In the control group, *S. mutans* developed dense, multilayered biofilms over time, with significantly increased thickness and surface coverage at 24 h compared to 6 h. Biofilms treated with 15ARG resembled the control at both time points, showing limited inhibitory effect on biofilm development. In contrast, the FBG-containing groups, including 15FBG, 15ARG + 15FBG and 7.5ARG + 7.5FBG, exhibited substantial suppression of biofilm formation, with visibly reduced bacterial colonization and extracellular matrix. Notably, both the composite groups (15ARG + 15FBG and 7.5ARG + 7.5FBG) not only showed the least bacterial colonization but also demonstrated apatite-like mineral deposition on the biofilm surfaces.

**Figure 4:**
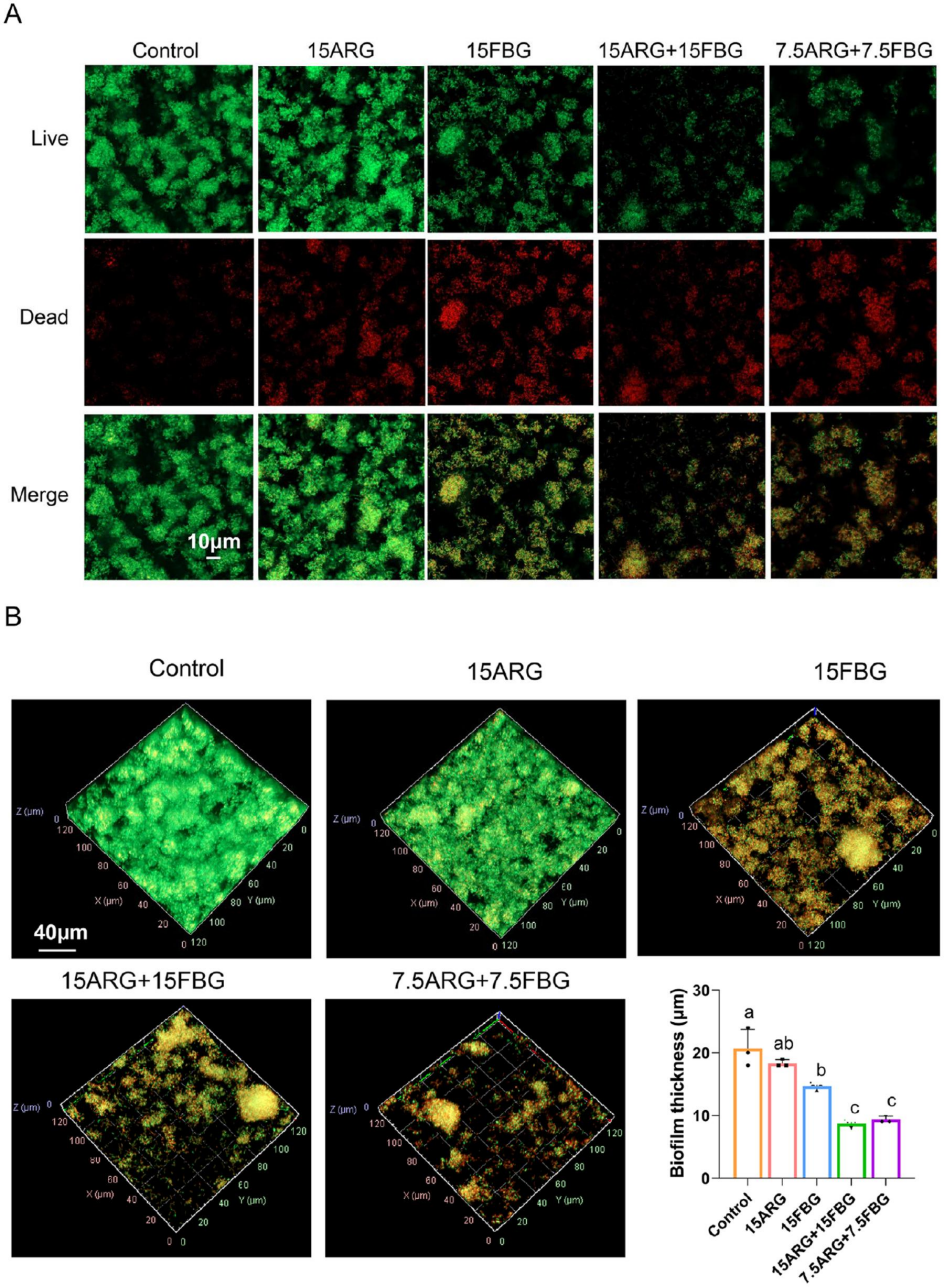
CLSM analysis of biofilm viability and structural integrity. (**A**) Representative 2D confocal images of *S. mutans* biofilms stained with live/dead dyes, scale bar = 10 μm. (**B**) Quantification of biofilm thickness derived from 3D reconstruction of *z*-stacked images.

CLSM imaging revealed significant disruption of *S. mutans* biofilms following 12 h treatment, as shown in Figure 4. Dense biofilm structures with predominantly live bacterial cells were observed in the control and 15ARG groups, indicating minimal disruption. In contrast, all FBG-containing treatments, particularly the composites 15ARG + 15FBG and 7.5ARG + 7.5FBG, resulted in marked increases in dead cell populations, as indicated by red fluorescence, and substantial reductions in biofilm thickness compared to the control and 15ARG alone (Figure 4B). Among all treatments, the two composites demonstrated the most pronounced biofilm disruption, with comparable levels of bacterial death and architectural degradation.

### ARG/FBG composites altered the bacterial ultrastructural morphology and elemental deposition

**Figure 5:**
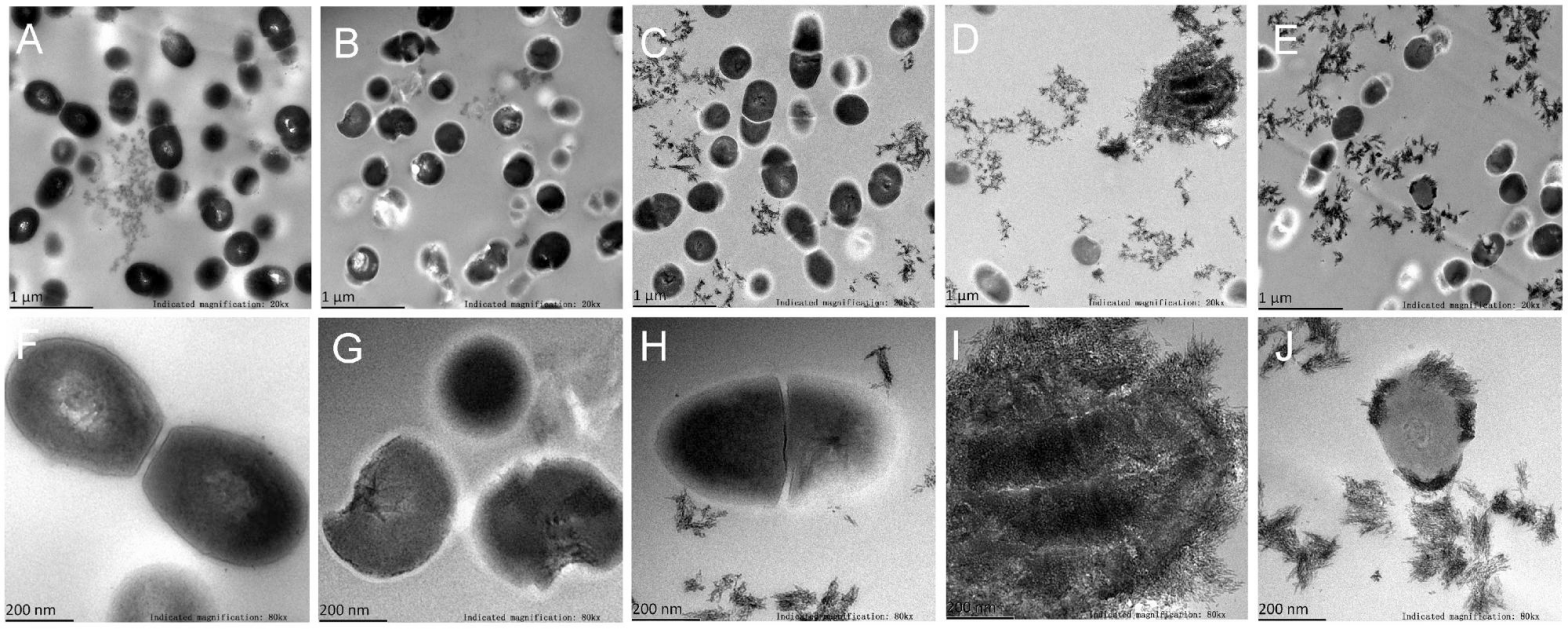
TEM ultrastructural analysis of *S. mutans* after 4 h treatment (**A, F**) control, (**B, G**) 15ARG, (**C, H**) 15FBG, (**D, I**) 15ARG + 15FBG, (**E, J**) 7.5ARG + 7.5FBG. Images acquired at 20 000× magnification (scale bar = 1 μm) and 80 000 × magnification (scale bar = 200 nm).

Representative TEM images of *S. mutans* after 4 h treatment are shown in Figure 5. In the control group, *S. mutans* cells displayed well-defined structures with intact membranes and evidence of binary fission (Figure 5A and F). In contrast, cells treated with 15ARG or 15FBG showed periplasmic halos and morphological variability, indicative of cellular stress or damage (Figure 5B, C, G and H). Degradation products from bioactive glasses were visible in the FBG-containing groups (15FBG, 15ARG + 15FBG and 7.5ARG + 7.5FBG) but not in the 15ARG group, confirming the physical presence and interaction of FBG with bacterial cells. In the composite-treated groups, TEM revealed a significant reduction in bacterial density, decreased electron density within the cytoplasm and suppression of binary fission, suggesting inhibited replication. Remarkably, the needlelike nanostructures were observed penetrating bacterial membranes, leading to cytoplasmic disorganization (Figure 5D, E, I and J).

**Figure 6:**
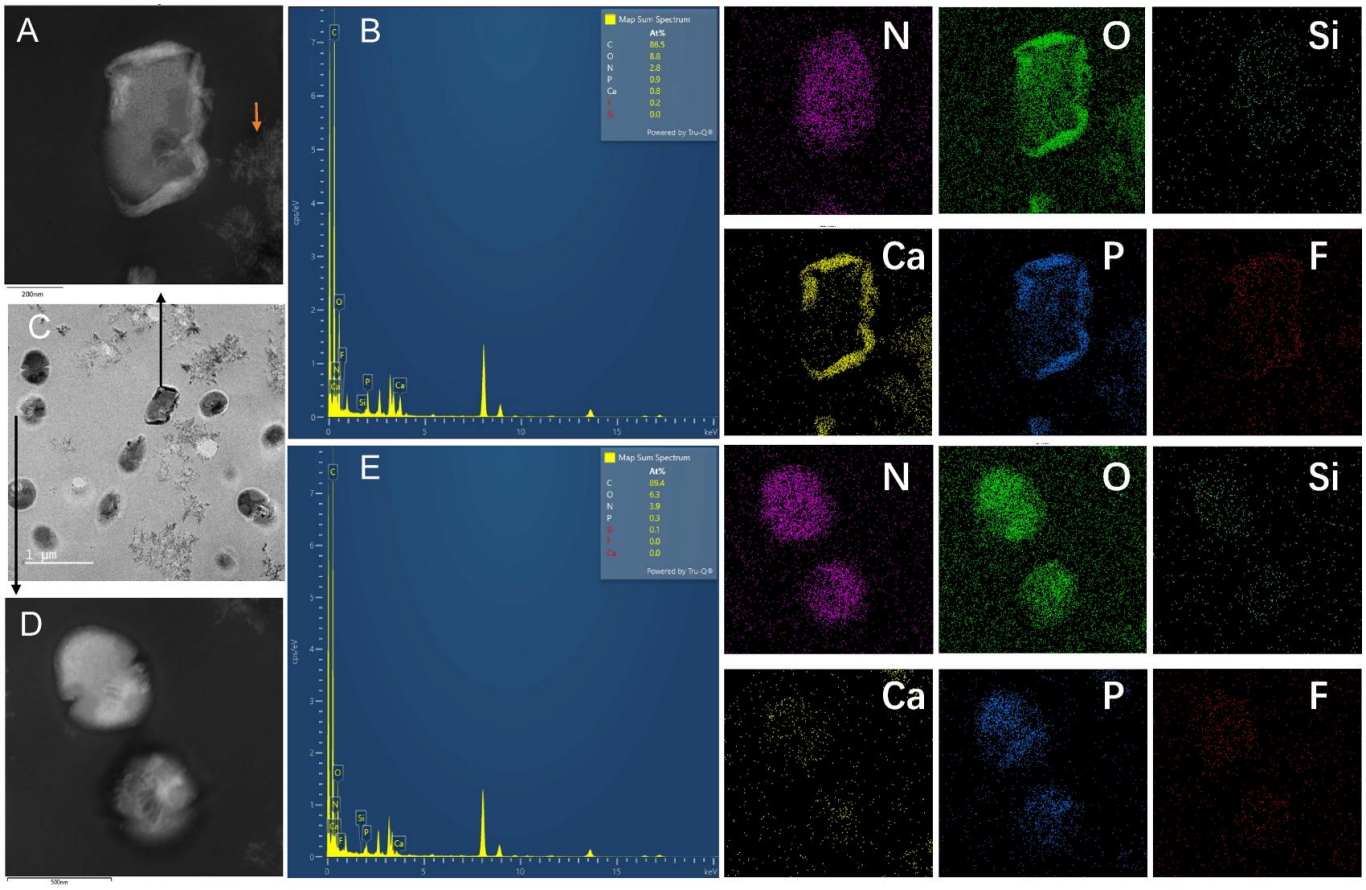
TEM-EDS element mapping of 15ARG + 15FBG-treated biofilms. (**A**) Bacterial cell penetrated by degradation products. (**B**) Corresponding EDS spectrum of (**A**). (**C**) Overview of analysed region. (**D**) Bacteria with less impact. (**E**) Corresponding EDS spectrum of (**D**). Abbreviations are nitrogen (N), oxygen (O), silicon (Si), calcium (Ca), phosphorus (P), fluorine (F).

EDS analysis confirmed that these needlelike structures embedded in cells were composed primarily of calcium (Ca), phosphorus (P) and fluorine (F) (Figure 6A and B). Co-localization of Ca/P/O signals was observed at bacterium-debris interfaces (arrow, Figure 6A). Conversely, TEM-EDS of untreated bacterial cells (Figure 6D and E) showed endogenous enrichment of carbon (C), nitrogen (N) and oxygen (O), consistent with the typical biomolecular composition of intact cells.

### Transcriptomic insights into the action of ARG/FBG composites against *S. mutans*

**Figure 7:**
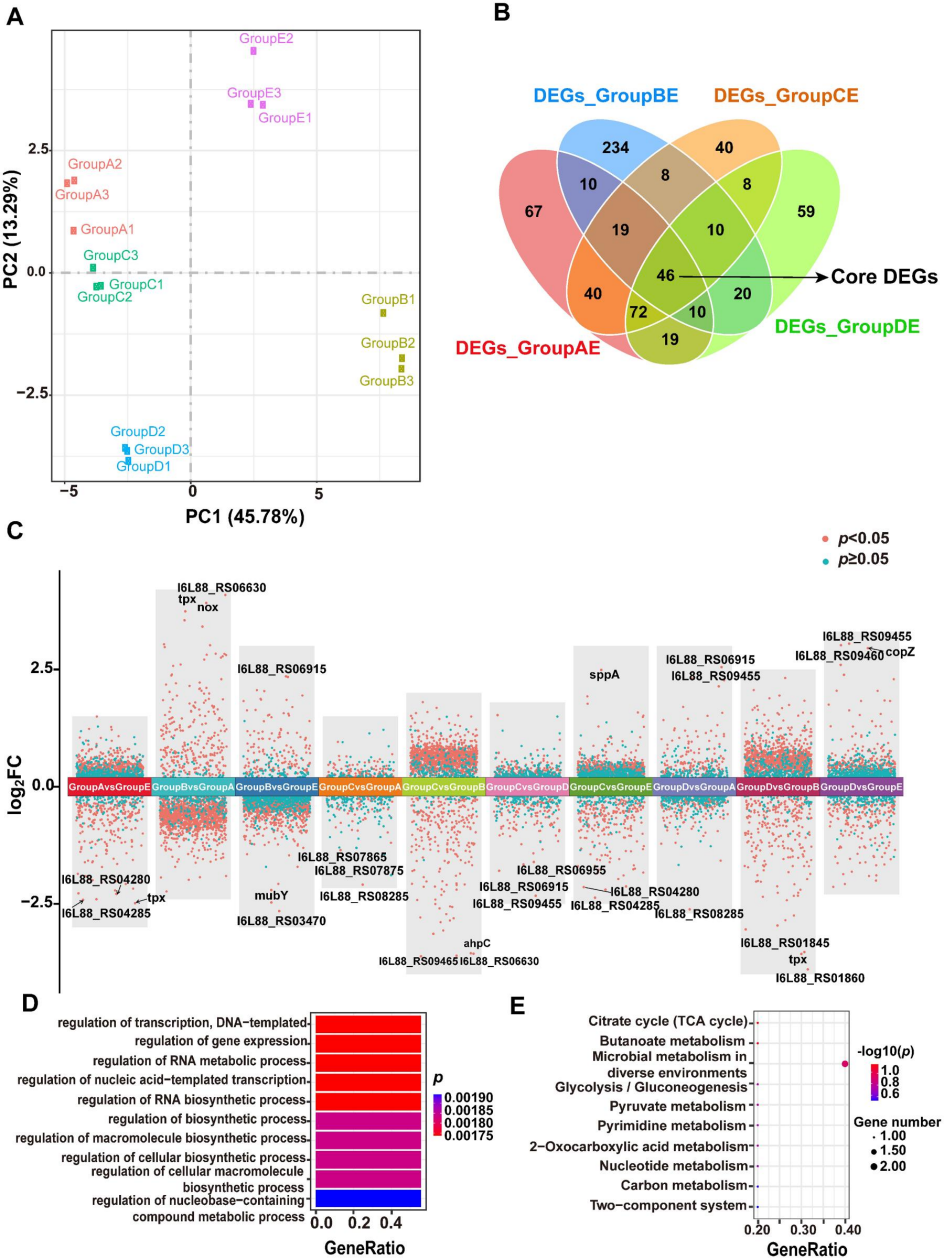
Quality control and transcriptomic profiling of *S. mutans* across five groups. (**A**) PCA plot, *P *< 0.05. (**B**) Venn diagram of overlapping core DEGs. (**C**) Volcano plots of DEGs. (**D**) GO enrichment analysis of DEGs. (**E**) KEGG pathway enrichment (top 10 pathways). Group definitions: 15ARG (**A**), 15FBG (**B**), 15ARG + 15FBG (**C**), 7.5ARG + 7.5FBG (**D**), control (**E**).

Quality control and analysis of RNA-seq data for the ARG/FBG composites against *S. mutans* activity are shown in Figure 7. Principal component analysis (PCA) demonstrated distinct clustering patterns among the treatment and control groups, accounting for 45.78% of the total variance in gene expression, while the tight clustering of biological replicates within each group validated the quality of the RNA-seq data (Figure 7A). Differentially expressed genes (DEGs) were identified using a false discovery rate (FDR)—adjusted *P* values of <0.05. A total of 46 core differentially expressed genes (DEGs) were identified across all treatment-versus-control comparisons (Figure 7B). Volcano plots highlight the top three most upregulated and downregulated genes in each group (Figure 7C). Gene ontology (GO) enrichment analysis showed significant DEG enrichment in categories such as transcription, gene expression and RNA metabolic process (Figure 7D). Kyoto Encyclopedia of Genes and Genomes (KEGG) analysis identified 10 significantly affected pathways, including citrate cycle (TCA cycle), butanoate metabolism, microbial metabolism in diverse environments, glycolysis/gluconeogenesis, pyruvate metabolism, pyrimidine metabolism, nucleotide metabolism, carbon metabolism and two-component system, suggesting that ARG and FBG exert broad regulatory effects on the metabolic activity of *S. mutans* (Figure 7E).

**Figure 8:**
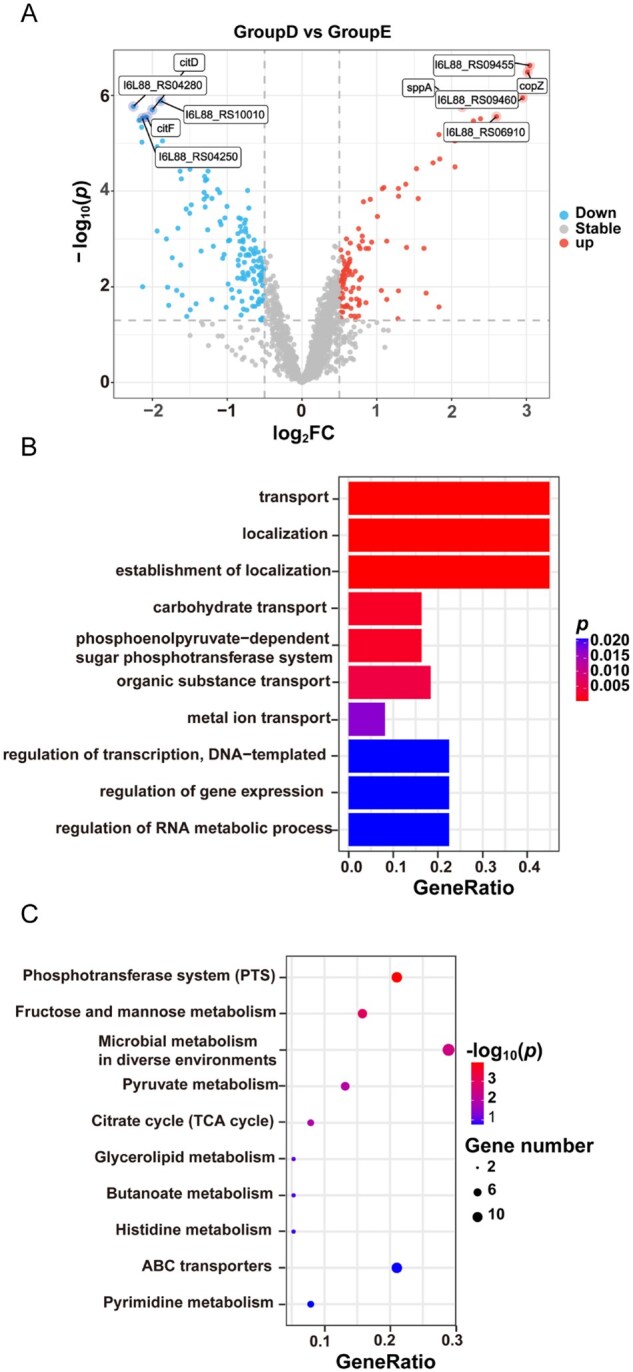
Transcriptomic signature of the 7.5ARG + 7.5FBG composite compared to the control. (**A**) Volcano plots of DEGs (FDR < 0.05 and |log_2_FC| ≥ 0.5). (**B**) GO enrichment. (**C**) KEGG pathway.

Further analysis comparing the 7.5ARG + 7.5FBG to the control identified 247 DEGs (FDR < 0.05, |log_2_FC| ≥ 0.5), including 138 downregulated and 109 upregulated genes (Figure 8A). These DEGs were predominantly related to the phosphotransferase system (PTS) and fructose and mannose metabolism, which likely play key roles in the observed enhanced antibacterial effects. GO enrichment highlighted functions related to transport and colonization (Figure 8B). KEGG pathway enrichment revealed significant modulation of multiple pathways, including PTS, fructose and mannose metabolism, microbial metabolism in diverse environments, pyruvate metabolism, citrate cycle, ABC transporters (Figure 8C). Similar pathway enrichments were observed for the 15ARG + 15FBG group (Supplementary Figure S3), indicating consistent metabolic responses to the ARG/FBG composites. Notably, PTS, fructose and mannose metabolism, ABC transporters and pyruvate metabolism, which are essential for biofilm formation and acid production, were coordinately regulated, suggesting disruption of *S. mutans* virulence by the ARG/FBG composites groups.

**Figure 9:**
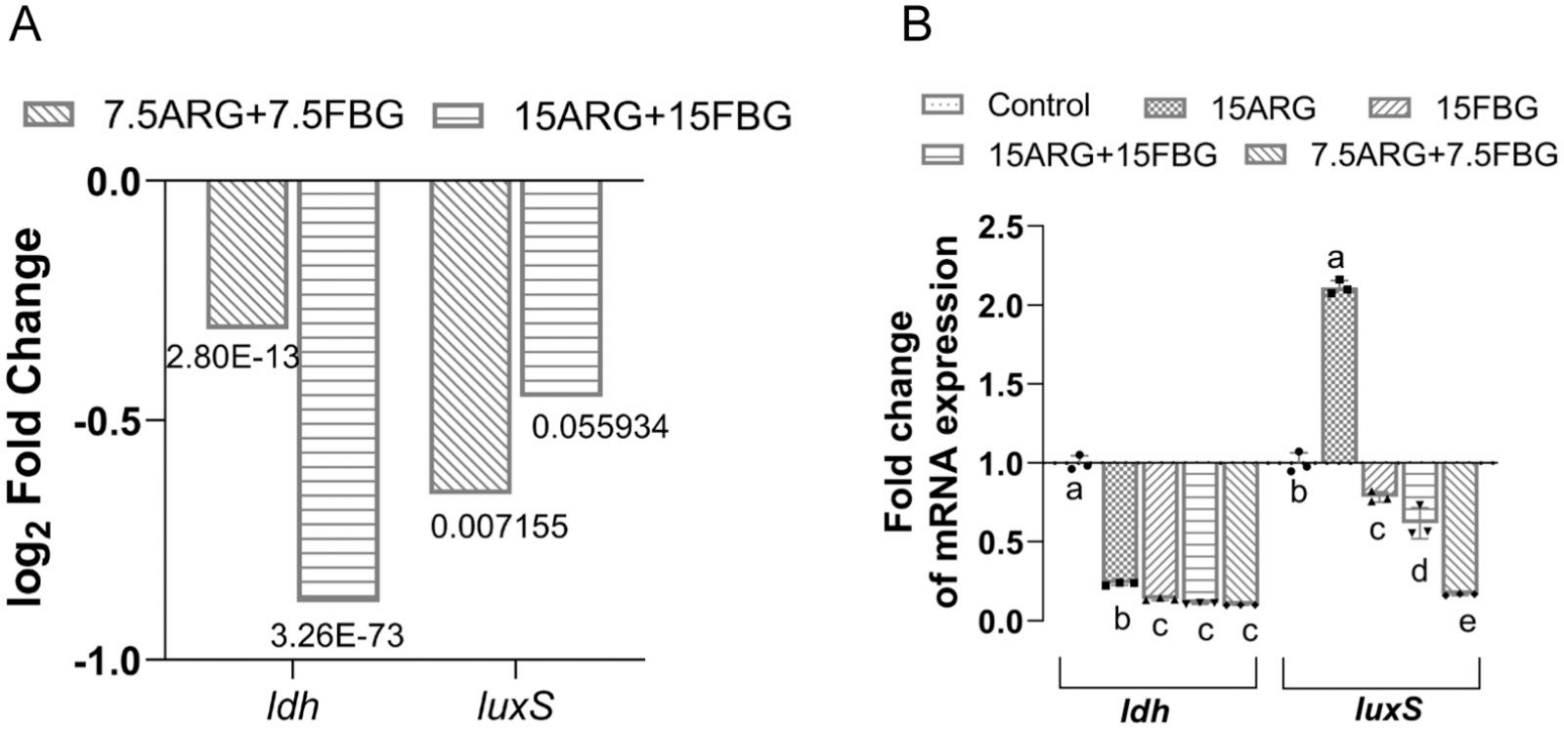
Validation of virulence gene suppression. (**A**) RNA-seq quantification of *ldh* and *luxS* expression. *P*-values indicate differences versus control. (**B**) RT-qPCR confirmation of *ldh* and *luxS* regulation across the treatment groups.

ARG and FBG elevated biofilm pH, while FBG showed significant biofilm disruption. These dual actions disrupt cariogenic biofilm development. In parallel, expression levels of *ldh*, which is involved in acidogenicity, and *luxS* that is critical for quorum sensing and biofilm formation were analysed. RNA-seq data showed downregulation of *ldh* in both the 7.5ARG + 7.5FBG and 15ARG + 15FBG composites compared to the control (Figure 9A), which was validated by RT-qPCR (Figure 9B). The 7.5ARG + 7.5FBG composite downregulated *luxS* expression according to the RNA-seq and RT-qPCR data, while 15ARG + 15FBG composite and 15FBG reduced *luxS* expression, 15ARG alone unexpectedly upregulated *luxS* (*P *< 0.05).

## Discussion

In this study, FBG exhibits strong antibiofilm activity with limited effects on planktonic growth, together with the known pH-modulating properties of ARG, supports a complementary strategy combining biofilm matrix disruption and alkali generation. Such an approach may attenuate cariogenic biofilm pathogenicity while preserving commensal-dominated communities, offering a more sustainable paradigm for caries prevention. Collectively, the design of ARG/FBG bioactive composites integrates antibiofilm disruption, pH modulation and sustained ion release, resulting in prolonged bioavailability compared with conventional fluoride, enhanced antibacterial efficacy over 45S5 bioactive glass and improved remineralization potential relative to ARG alone.

Sub-MICs of ARG and FBG inhibited *S. mutans* growth in a dose-dependent manner, with the composite MIC determined at 7.5 mg/mL ARG + 7.5 mg/mL FBG. This optimal formulation demonstrated excellent biocompatibility and was selected for further experiments using a simulated biofilm model to explore effects and mechanisms of the composites.

Pathogenic biofilm formation drives dental [45], disrupting biofilm structure and function is critical for effective caries prevention [46]. *S. mutans* biofilms rely on the EPS matrix, composed of glucans, proteins and extracellular DNA (eDNA), which supports adhesion, structural integrity and virulence [47]. A previous study showed that NaF reduced EPS production, while the addition of ARG provided minimal additional suppression [28], suggesting limited anti-biofilm activity of ARG. This could be attributed to the nitrogenous nature of ARG, which may promote protein retention within the biofilm, as well as the dual role of eDNA and polysaccharides in reinforcing biofilm structure. In contrast, FBG effectively disrupted biofilm architecture, likely through sustained fluoride release that interferes with glycolysis and increases membrane proton permeability and osmotic/alkaline stress in the biofilm [48–51], although its activity against planktonic *S. mutans* is limited. ARG alters microbial ecology toward a health-associated profile, buffers pH and efficiently inhibits planktonic *S. mutans* growth [25, 52]. Notably, ARG/FBG composites exhibited potent antibacterial activity and biofilm disruption of *S. mutans* for 2–24 h treatment period, suggesting an enhanced and complementary interaction, aligning with findings on adding ARG into 5% NaF enhancing ecological balance by Bijle *et al.* [28]. Interestingly, 7.5ARG + 7.5FBG composite exhibited comparable or slightly superior antibacterial efficacy to the 15ARG + 15FBG formulation and superior efficacy to the 3.75ARG + 3.75FBG formulation. These findings support the 7.5ARG + 7.5FBG composite as a low-dose, biosafe and sustainable strategy for caries prevention, likely due to an optimal balance in ion release, pH modulation and microbial suppression.

Disruption of bacterial cell walls compromises membrane integrity and leads to osmotic imbalance and cell lysis [53]. Consistent TEM and CLSM observations indicated reduced *S. mutans* viability following ARG/FBG composites treatment, likely due to the combined suppression of proliferation and metabolic activity by ARG and fluoride [54]. TEM-EDS mapping indicated fluorapatite-like mineral deposits within or on bacterial surfaces. Such *in situ* mineralization may exert antibacterial effects by damaging membranes, obstructing nutrient transport and inhibiting metabolic activity, while fluoride, calcium and phosphate jointly create a hostile microenvironment for cariogenic bacteria [10, 55, 56]. Moreover, apatite-like deposition within the biofilm matrix, observed by SEM and TEM-EDS may disrupt EPS architecture and provide bioavailable ions for enamel remineralization [57, 58]. Collectively, these findings indicate that *in situ* mineral deposition enables the ARG/FBG composites to simultaneously suppress cariogenic biofilm and support hard tissue repair.

RNA-seq analysis provided mechanistic insights into the antibacterial action of ARG/FBG composites against *S. mutans*. Genes involved in carbohydrate transport and metabolism, particularly the PTS system and fructose and mannose metabolism pathways, were significantly regulated, indicating impaired sugar uptake, energy metabolism and EPS synthesis [45, 59–61]. In parallel, expression of lactate dehydrogenase (*ldh*), a key enzyme in lactic acid production which was typically upregulated in acidic and pathogenic microenvironments, was strongly suppressed, consistent with elevated local pH resulting from ARG-derived ammonia and FBG-induced alkalization [62–64]. Moreover, the quorum sensing regulator *luxS*, critical for biofilm maturation and virulence, was downregulated by FBG and ARG/FBG composites but not by ARG alone, aligning with reduced biofilm formation [57, 58, 65, 66]. The obtained results support that the ARG/FBG composites might exert antibacterial and antibiofilm effects through the following aspects: inhibition of carbohydrate uptake and metabolism, suppression of acid production via *ldh* downregulation, disruption of cell–cell communication by interfering with *luxS* and reduction of virulence gene expression and EPS formation.

The engineered ARG/FBG bioactive composites outperformed single components in antibacterial and antibiofilm efficacy and biocompatibility. They acted enhanced consistent with a complementary mechanism involving: (1) microenvironment alkalinization by ARG metabolism (ammonia) and FBG dissolution (OH^−^ release), suppressing acidogenesis, (2) disruption of bacterial membranes and glycolysis by FBG-derived ions, destabilizing *S. mutans* colonization [29], (3) the ecological regulation mediated via ARG-fluoride [67], (4) suppression of virulence genes associated with carbohydrate uptake and metabolism disrupted. In addition, our prior demineralization studies demonstrated that the 7.5ARG + 7.5FBG formulation provides superior anti-demineralization benefits [28], while lower fluoride content may reduce the risk of fluorosis and enhance formulation safety. The potential formation of ARG–F–Ca complexes may further prolong antibacterial activity by facilitating fluorapatite delivery and retention within biofilms. These findings position the ARG/FBG composite as a promising bioactive additive for dental products, such as toothpaste or mouthwash for caries prevention, capable of balancing efficacy, safety and cost-effectiveness. However, this study has limitations. The model employs a single bacterial species (*S. mutans*) *in vitro*, not capturing the complexity of multi-species oral biofilms or host factors. Future work requires validation in multi-species biofilm models, animal caries studies and long-term safety assessments to confirm clinical potential. The precise physicochemical nature of the observed intracellular mineral deposits also warrants further investigation.

## Conclusions

In summary, ARG primarily targets planktonic growth, while FBG disrupts biofilms. The engineered ARG/FBG bioactive composites combine the benefits of both ARG and FBG by creating an alkalizing microenvironment, forming fluorapatite-like phase to obstruct nutrient transport and regulating key pathways involved in acid–base balance, carbon metabolism and biofilm development at the transcriptomic level, thereby exerting potent dual-pathway suppression of cariogenic bacterial planktonic growth and biofilm formation. The 7.5ARG + 7.5FBG formulation not only enhanced antibacterial potency but also improved biosafety and optimized fluoride bioavailability, enhancing safety. The ARG/FBG bioactive composites are promising additives for caries prevention. Future studies will focus on *in vivo* validation using rodent caries models, long-term biosafety evaluations and more in-depth mechanistic studies.

## Supplementary Material

rbag058_Supplementary_Data
