# Supplementary material for: A novel dual-targeting strategy to suppress cariogenic bacteria and biofilms with engineered ARG/FBG bioactive composites
Source: Regen Biomater. 2026 Mar 20;13:rbag058. doi: 10.1093/rb/rbag058 (PMC13089478; doi:10.1093/rb/rbag058)
Supplement: rbag058_Supplementary_Data [file rbag058_supplementary_data.zip › Supplementary data RB-2025-663.docx]

**Supplementary data**

**Table S1.** Optical density (OD₆₀₀ nm) values of *S. mutans* before and after 24 h treatment with ARG and FBG alone or their composite

| Groups  (mg/mL) | ARG | | FBG | | 1/2ARG+1/2FBG | | *S.mutans*+  BHI control | | BHI control | |
| --- | --- | --- | --- | --- | --- | --- | --- | --- | --- | --- |
|  | 0 h | 24 h | 0 h | 24 h | 0 h | 24 h | 0 h | 24 h | 0 h | 24 h |
| 0.9375 | 0.05 | 0.661 | 0.539 | 1.021 | 0.122 | 0.836 | 0.074 | 0.757 | 0.073 | 0.08 |
|  | 0.05 | 0.936 | 0.178 | 0.741 | 0.152 | 0.765 | 0.069 | 0.776 | 0.072 | 0.079 |
|  | 0.049 | 0.92 | 0.164 | 0.565 | 0.119 | 0.728 | 0.072 | 0.832 | 0.073 | 0.081 |
| 1.875 | 0.049 | 0.941 | 0.291 | 0.815 | 0.152 | 0.746 |  |  |  |  |
|  | 0.049 | 0.891 | 0.259 | 0.854 | 0.205 | 0.868 |  |  |  |  |
|  | 0.049 | 0.882 | 0.282 | 0.884 | 0.169 | 0.885 |  |  |  |  |
| 3.75 | 0.051 | 0.713 | 0.587 | 1.065 | 0.429 | 1.004 |  |  |  |  |
|  | 0.049 | 0.813 | 1.212 | 1.423 | 1.056 | 1.056 |  |  |  |  |
|  | 0.049 | 0.815 | 0.416 | 1.052 | 0.391 | 1.087 |  |  |  |  |
| 7.5 | 0.048 | 0.124 | 1.615 | 1.61 | 0.678 | 0.678 |  |  |  |  |
|  | 0.051 | 0.082 | 1.673 | 1.698 | 0.772 | 1.349 |  |  |  |  |
|  | 0.05 | 0.083 | 1.584 | 1.666 | 1.426 | 1.512 |  |  |  |  |
| 15 | 0.045 | 0.084 | 1.8 | 1.761 | 1.52 | 1.515 |  |  |  |  |
|  | 0.051 | 0.083 | 1.51 | 1.471 | 1.66 | 1.625 |  |  |  |  |
|  | 0.049 | 0.089 | 1.709 | 1.745 | 1.078 | 1.091 |  |  |  |  |
| 30 | 0.048 | 0.083 | 1.828 | 1.758 | 1.648 | 1.623 |  |  |  |  |
|  | 0.05 | 0.083 | 1.911 | 1.908 | 1.796 | 1.752 |  |  |  |  |
|  | 0.05 | 0.084 | 1.901 | 1.866 | 1.458 | 1.466 |  |  |  |  |
| 60 | 0.049 | 0.079 | 2.176 | 2.09 | 1.961 | 1.914 |  |  |  |  |
|  | 0.049 | 0.083 | 2.241 | 2.166 | 1.833 | 1.774 |  |  |  |  |
|  | 0.049 | 0.085 | 2.149 | 2.11 | 1.916 | 1.878 |  |  |  |  |


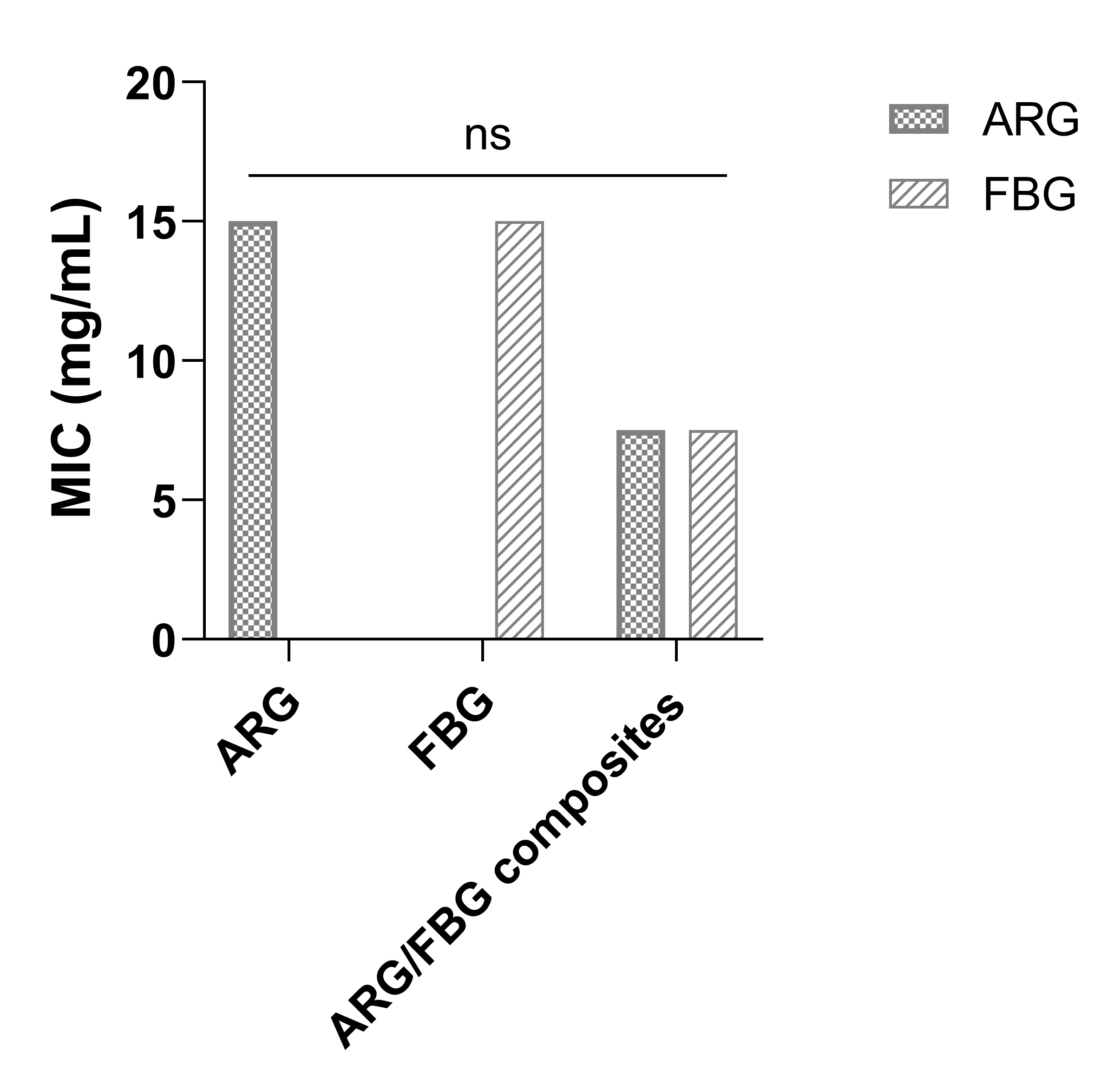


**Figure S1.** The MIC of ARG, FBG and ARG/FBG composites against *S. mutans*. ns indicates no significant differences.


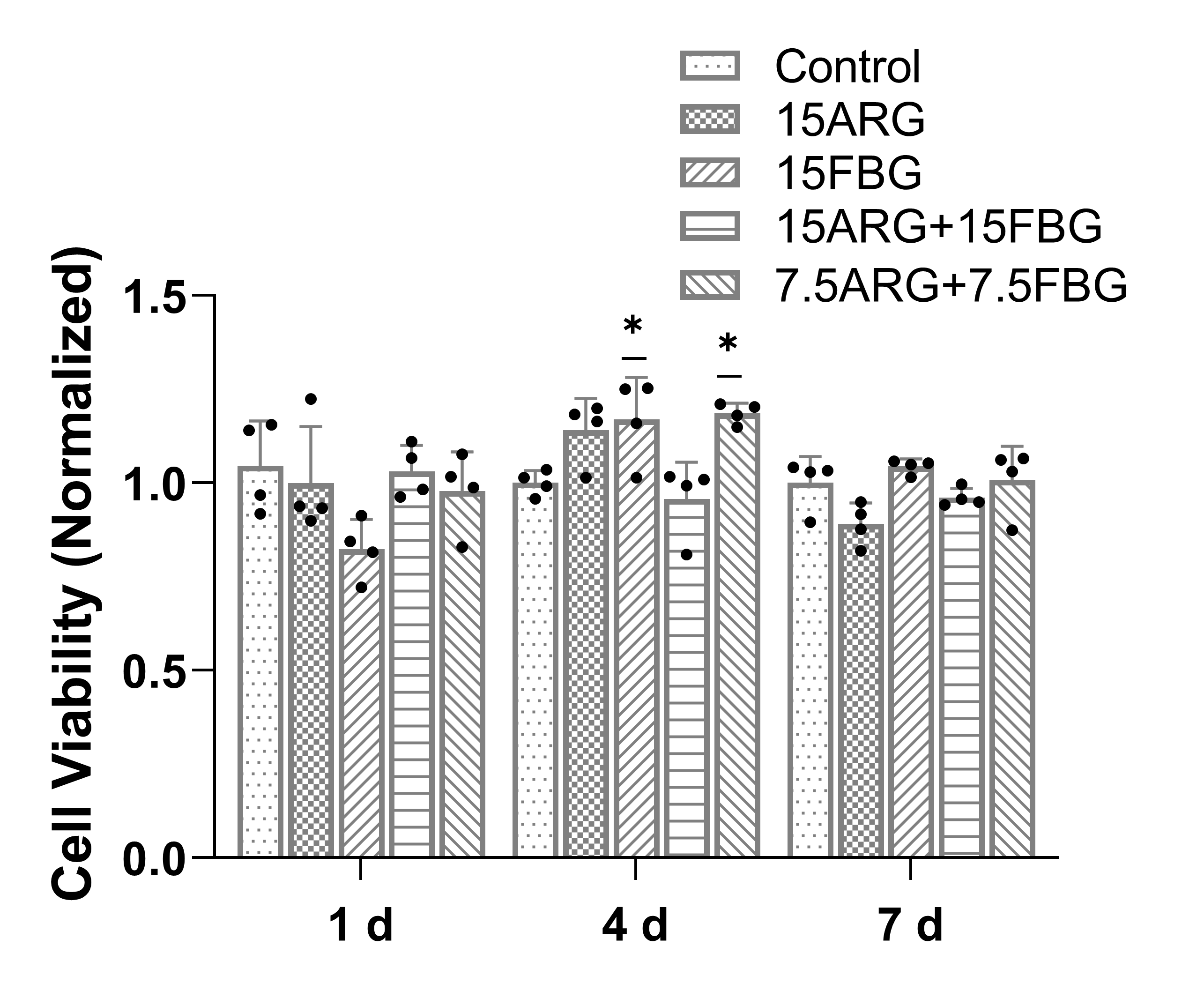


**Figure S2.** Viability of MC3T3-E1 cells exposed to ARG and FBG extracts. Significant differences **p* < 0.05 versus Control.


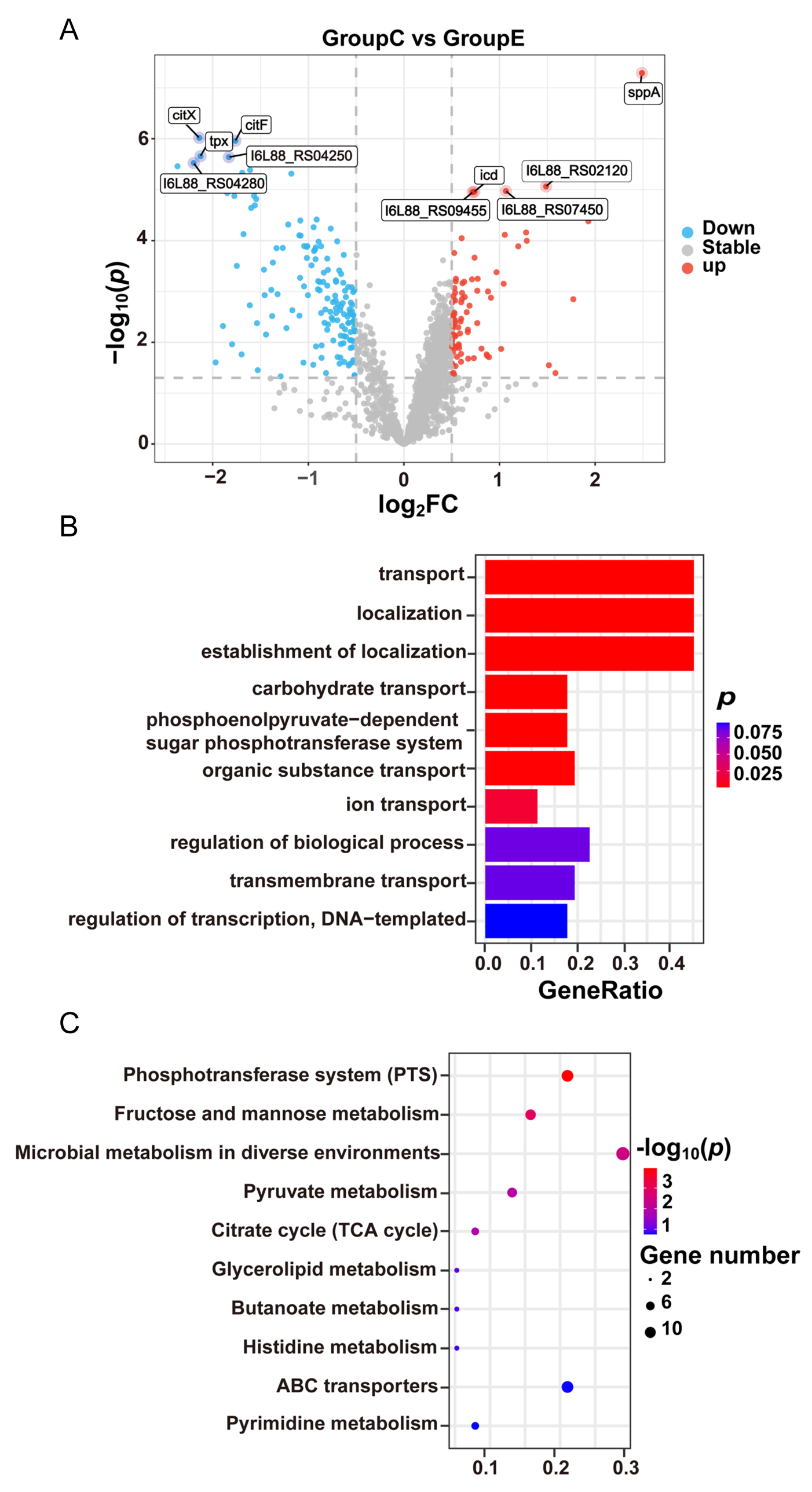


**Figure S3.** Transcriptomic signature of 15ARG+15FBG compared to Control. (A) Volcano plots of DEGs (FDR < 0.05 and |log_2_ FC| ≥ 0.5). (B) GO enrichment. (C) KEGG pathway.
